# Nature Meets Science: The Role of Food-Grade Oils and Green Excipients in Pharmaceutical Nanoemulsion Formulation

**DOI:** 10.3390/ma19071294

**Published:** 2026-03-25

**Authors:** Fabrizio Villapiano, Maria Capuano, Federica D’Aria, Concetta Giancola, Virginia Campani, Giuseppe De Rosa, Marco Biondi, Laura Mayol

**Affiliations:** 1 Dipartimento di Farmacia, Università degli Studi di Napoli Federico II, Via Domenico Montesano 49, 80131 Naples, Italy; fabrizio.villapiano@unina.it (F.V.); maria.capuano2@unina.it (M.C.); federica.daria@unina.it (F.D.); giancola@unina.it (C.G.); gderosa@unina.it (G.D.R.); laumayol@unina.it (L.M.); 2 Dipartimento di Scienze della Vita, della Salute e delle Professioni Sanitarie, Università degli Studi Link Campus University, 00165 Roma, Italy; v.campani@unilink.it; 3 Centro di Ricerca Interdipartimentale sui Biomateriali (CRIB), Università degli Studi di Napoli Federico II, Piazzale Vincenzo Tecchio 80, 80125 Naples, Italy

**Keywords:** green nanoemulsions, food-grade oils, natural surfactants, drug delivery, physicochemical stability, sustainable formulations

## Abstract

Nanoemulsions are pivotal carriers which are increasingly adopted as carriers for poorly soluble active molecules. This review provides a critical overview of ‘green’ nanoemulsions, which are systems based on renewable, biodegradable, and non-toxic components and/or using sustainable production techniques. We here focus on the role of food-grade oils (including poly-unsaturated fatty acid-rich sources) and green excipients, with special attention on the interfacial properties of biosurfactants such as proteins, polysaccharides, and small-molecule surfactants. This review provides a critical overview of the formulation principles, interfacial phenomena, and physicochemical stability of green nanoemulsions, with reference to topical and pharmaceutical applications. The performance of nanoemulsions as delivery systems for bioactive lipids, essential oils, vitamins, carotenoids, phenolic compounds, and conventional drugs is examined through representative case studies. Known limitations, including oxidative instability, compositional variability, and difficulties in large scale production, are analyzed along with future opportunities in multifunctional formulations and sustainable processing. Overall, green nanoemulsions emerge as promising next-generation platforms for safe, effective, and environmentally friendly drug delivery.

## 1. Introduction

**Figure 1:**
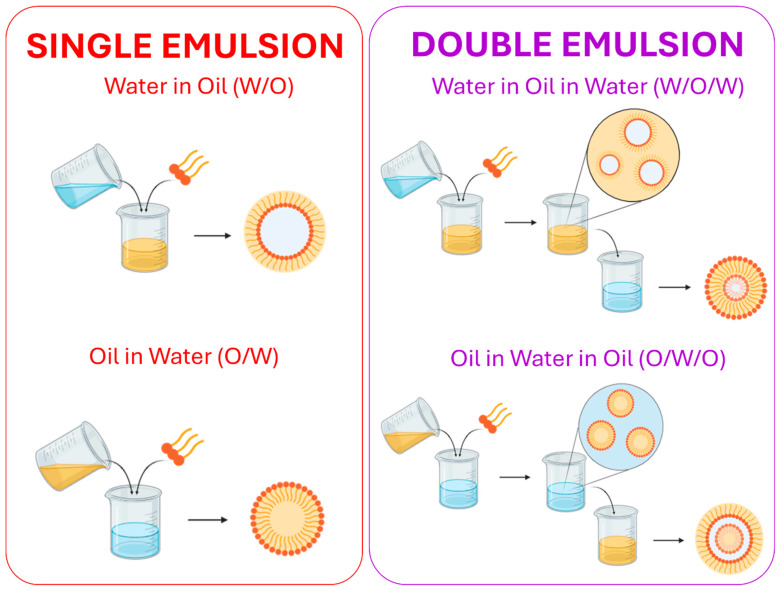
Structure of single and double nanoemulsions. Water-in-oil (W/O) and oil-in-water (O/W) nanoemulsions (**left**). Water-in-oil-in-water (W/O/W) and oil-in-water-in-oil (O/W/O) double emulsions (**right**). Created with BioRender.com.

Nanoemulsions (NEs) are liquid heterogeneous systems, typically in an oil-in-water (O/W) or water-in-oil (W/O) form (Figure 1), characterized by an ultra-fine droplet diameter, ranging from tens to hundreds of nm [1,2].

NEs are described by the volume ratio between the phases (Φ), stabilized using one or more appropriate surfactants [3]. The preparation of an emulsion requires mechanical work to disperse the internal phase into the external one, which leads to a strong increase in the total surface area of the system [3,4,5]. Once the mechanical action is completed, the emulsions are thermodynamically unstable and spontaneously tend to phase separation to minimize the free energy of the system (ΔG), as will be discussed in Section 7 [3].

In the most frequent case of O/W NEs, in accordance with Bancroft’s rule, water-soluble surfactants are used since they are more soluble in the continuous phase [6,7]. The stability against coalescence in O/W NEs is widely attributed to interfacial films and repulsive interactions arising from the polar headgroups [8,9]. Consequently, O/W NEs can be formed using relatively high internal phase volume ratios [7,10]. Phase inversion can occur when comparable volumes of the oil and water phase are mixed together (roughly for Φ ranging between 0.5 and 0.7 and higher, depending on the formulation conditions) [6,7,10,11,12].

A significant challenge in drug delivery is that 40% of currently marketed drugs and a higher percentage of new chemical entities are poorly water-soluble, and this severely limits their dissolution rate and subsequent oral bioavailability. NEs, particularly the O/W type, emerge as a remarkable strategy to overcome this limitation by efficiently solubilizing hydrophobic active pharmaceutical ingredients (APIs), thereby improving their solubility and, accordingly, their systemic bioavailability [13]. Furthermore, NEs often exhibit optical transparency, and a high surface area, intrinsically linked to nanoscale size [1,14,15].

NEs are characterized by high encapsulation efficiency, loading capacity, and feasibility of preparation. Thus, they have been extensively explored for the encapsulation of both food-based bioactives and drugs [16,17], offering the possibility to overcome the limitations of conventional pharmaceutical formulations [18,19].

It should be noted, however, that NEs in the pharmaceutical field also have some limitations. For instance, APIs with a high melting point are not suitable for loading into these systems. Furthermore, if they are intended for human use, NEs must be made of non-toxic (generally regarded as safe, GRAS) substances. This necessity, combined with the energy consumption of high-energy preparation methods, raises significant questions about the overall sustainability and environmental impact of NE production [18,19,20,21].

This perspective addresses the recent advances in the design and development of “green” NEs, that align with sustainability and eco-friendly principles. Specifically, green NEs are composed of natural, renewable, and biodegradable components, such as food-grade oils, and plant-derived surfactants. Furthermore, they are formulated with sustainable fabrication processes and/or green synthesis methods, which avoid toxic solvents and harmful chemicals, enhancing biocompatibility and minimizing the environmental impact. Furthermore, the adoption of green excipients and processes supports regulatory compliance and consumer demand for environmentally responsible products [22].

Within this broader context, the present review is primarily focused on pharmaceutical NE formulation within a materials and physicochemical framework. References to food and cosmetic applications are included to highlight shared formulation principles and broader technological relevance.

## 2. Food-Grade Oils in Nanoemulsions

The formulation of NEs requires careful selection of excipients in both the aqueous and oil phases. Indeed, the organic (oily) phase largely determines the solubility, protection, and bioavailability of lipophilic compounds. From a green perspective, food-grade oils offer a safe and biocompatible solution, capable of serving both as carriers and therapeutic adjuvants [23].

Quality and regulatory requirements for food-grade oils depend on their intended use, i.e., food, cosmetic, or pharmaceutical. While food and cosmetic applications are governed by their respective safety regulations, food-grade oils can be employed in pharmaceutical formulations if they meet pharmacopeial quality standards, in terms of purity, compositional consistency, and reproducibility. In this context, specifications vary according to the route of administration (oral, topical, parenteral, etc.), and therefore suitability must be evaluated on a case-by-case basis.

**Figure 2:**
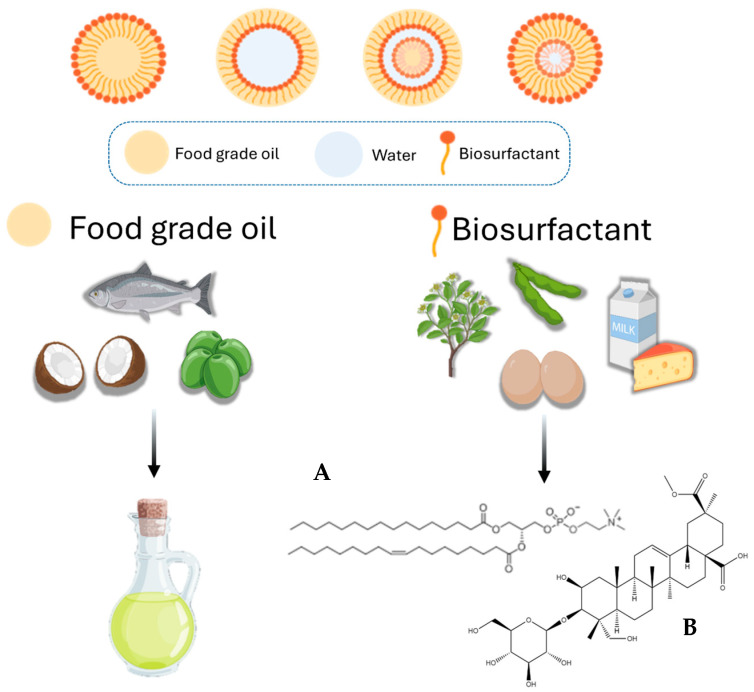
Examples of food-grade excipients used in green nanoemulsions: food-grade oils (e.g., fish, coconut, avocado oils) and natural biosurfactants (e.g., plant-derived compounds, egg and milk proteins) such as phospholipids (**A**) and saponin (**B**). Created with BioRender.com.

So far, a number of food ingredients and additives, including bioactive lipids, vitamins and flavorings, have been used in NE formulation, as shown in Figure 2 and summarized in Table 1 [24,25].

Essential polyunsaturated (PUFA) fatty acids, such as omega-3 oils and α-linolenic acid, are the main bioactive lipids [24,25], which reportedly possess substantial health benefits, especially for the neuroplasticity of nerve membranes and synaptogenesis [26]. However, bioactive lipids are highly unstable against oxidation and show persistent odor thresholds. Therefore, their encapsulation in NEs can reduce autoxidation and mask unpleasant tastes [27].

Lipophilic vitamins are biologically sensitive substances, displaying marginal chemical stability and water solubility [28]. For instance, Vitamins A, and Vitamins E are easily oxidized, particularly when exposed to light, heat, light, and metal ions. Furthermore, visible and fluorescent light possessed the ability to dramatically alter the vitamin K structure. They are usually fabricated to improve their chemical stability, solubility and oral bioavailability. As reported by Lv et al., NEs containing vitamin E were fabricated by dual-channel microfluidizer, using corn oil as a carrier oil resulting in an improved vitamin bioavailability (53.9%) [29]. In another example, a curcumin-containing NE was produced. This molecule is a polyphenol extracted from the rhizome of *Curcuma longa Linn*, endowed with anti-inflammatory, antioxidant, and antimicrobial properties. Curcumin applicability is limited due to its negligible aqueous solubility and consequently very low bioavailability [30]. These limitations were overcome by preparing a phospholipid-stabilized NE, whose organic phase consists of curcumin in flaxseed oil, showing a drastic increase in its bioavailability in rats [31].

## 3. Green Surfactants in Nanoemulsion Formulation

In NEs, surfactants drive the formation and stabilization of the colloidal structure. By adsorbing at the oil–water interface, they lower interfacial tension and facilitate the generation of finely dispersed droplets, ultimately enhancing the physical stability of the NE [32,33]. However, increasing concerns regarding the ecological and health impacts of synthetic surfactants are stimulating the development of “green” alternatives, such as natural surfactants, which offer a lower environmental footprint compared to conventional options [34]. In this context, the term “green” refers to materials derived from renewable sources, showing improved biocompatibility, biodegradability, lower toxicity, and reduced environmental impact compared to conventional options [34].

As with any industrial material, the production of green excipients may still involve environmental burdens depending on source, extraction method, and scale. Nevertheless, bio-based excipients are generally associated with biodegradability properties and reduced toxicity compared to conventional synthetic surfactants, which supports their increasing adoption in sustainable formulations [35,36]. These considerations further motivate the shift towards more sustainable surfactant systems [37]. Green surfactants include several molecular classes, such as phospholipids, proteins, saponins, and polysaccharides, which ensure the physical stability of nanoscale dispersions through steric and/or electrostatic mechanisms, similarly to conventional chemical surfactants (Figure 2).

Phospholipids (PLs) are a major class of amphiphilic molecules widely employed as natural emulsifiers owing to their distinctive structural organization. They consist of a glycerol backbone esterified with two non-polar fatty acid chains and a polar phosphate group, which is further linked to various polar organic moieties. This structural arrangement provides PLs with their characteristic amphiphilic nature, thus reducing the interfacial tension [33].

Proteins are being increasingly studied as natural emulsifiers due to their biodegradability, versatility and amphiphilic nature, which allow them to adsorb at the oil–water interface and form stable interfacial layers [38].

Saponins are a class of natural surfactants characterized by an amphiphilic structure composed of a lipophilic aglycone (typically a triterpenoid or steroid) and a hydrophilic glycone portion containing one or more sugar moieties [21,39].

Polysaccharides are not amphiphilic molecules and therefore cannot be classified as true emulsifiers; for this reason, they are often used in combination with other surfactants and co-surfactants. Nevertheless, they can act as effective stabilizing agents due to their hydrophilicity, highly branched structure, and high molecular weight, which provide thickening and gelling capabilities. These properties increase the viscosity of the continuous phase and create a steric barrier that limits destabilizing mechanisms [34]. Furthermore, they can form a compact hydrophilic shell around the oil droplets, providing strong three-dimensional steric repulsion [23]. Table 2 reports an overview of green surfactants, classified according to their different physico-chemical parameters.

Gao et al. [40] developed NEs based on fractionated coconut oil using both natural and synthetic surfactants, with the aim of comparing their performance. PLs such as whey protein isolate and soy lecithin, in addition to the widely employed tea saponin, were employed as natural surfactants, whereas Tween 80 served as synthetic reference. The authors examined how emulsifier type and concentration, as well as pH, ionic strength, and heat treatment, influenced droplet size, ζ-potential, and stability. Tea saponin and soy lecithin generated smaller droplets than whey protein isolate, and also showed emulsifying performance comparable to Tween 80, likely due to a combined electrostatic and steric stabilization. The stability of the resulting NEs was influenced by many factors: as for whey protein isolate, it was affected by acidic pH and high temperature; for soy lecithin, by low pH and high ionic strength, while tea saponin provided the most robust stability across tested conditions and maintained long-term stability even at 27 and 50 °C. Overall, tea saponin emerged as the most effective natural emulsifier, offering stability and droplet size control comparable to Tween 80 and superior to protein or phospholipid based emulsifiers.

Also Najmeh Oliyaei et al. [41] compared natural and synthetic surfactants. They investigated the potential of stabilizing fucoxanthin-loaded NE using natural emulsifiers. Specifically, they compared Tween 80 with protein-based natural emulsifiers (sodium caseinate) and polysaccharide-based natural emulsifiers (fucoidan and gum Arabic). The study evaluated NE stability, particle size, ζ-potential, encapsulation efficiency, morphology, and in vitro release. The results indicated that, although fucoidan and gum Arabic achieved higher encapsulation efficiency and fucoxanthin release than sodium caseinate and Tween 80, they exhibited poor stabilizing properties. This highlighted the importance of combining polysaccharides with other natural emulsifiers or with a small amount of synthetic emulsifiers, to improve NE stability.

## 4. Formulation Techniques Using Green Materials

The basis of NE formation consists of the generation of minute droplets of the dispersed phase into the surrounding continuous phase [3,12]. Based on the selection of green oils and excipients discussed above, NEs can be formulated through different preparation techniques, which are outlined in this section.

Over the decades, the adopted techniques have been divided into two main groups: high-energy and low-energy methods (Table 3). The choice of the method depends on several factors, such as the nature of the components, the desired mean droplet size distribution, and the scale of operation (i.e., laboratory development or industrial production).

In high-energy emulsification methods, the driving forces are the high mixing speed and the shear stress generated, which disrupt micro-droplets into nano-droplets, through a top-down approach. These require specialized equipment such as high-pressure homogenizers, sonicators, and microfluidizers, which are expensive in terms of both purchase cost and energy consumption. In stark contrast, low-energy emulsification methods do not require specialized equipment, since the driving force of emulsion formation is rooted in the physicochemical properties of components. The process relies on the intrinsic energy stored within the system, enabling the components to spontaneously self-assemble into an emulsion, through a bottom-up approach, typically achieved by modulating environmental parameters such as temperature and pH, or by gradually increasing the concentration of the components over time.

### 4.1. High Energy Emulsification

#### 4.1.1. High Pressure Homogenization

**Figure 3:**
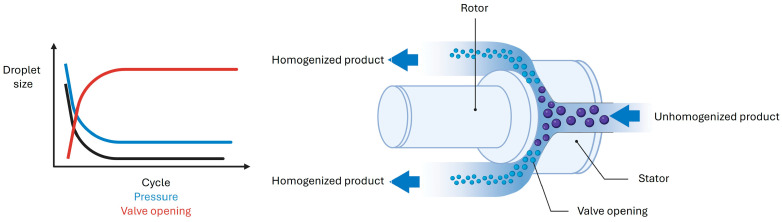
Nanoemulsion formation by high pressure homogenization. Created with BioRender.com.

High-pressure homogenization employs a specialized apparatus that applies substantial energy input to reduce the microdroplets in nanodroplets. During the process, the dispersion is forced to pass through a homogenization stage consisting of a rapidly rotating rotor positioned in close proximity to a fixed stator. The combination of high-rotational speed and the resulting increase in surface area generates disruptive forces that fragment the droplets. Many apparatuses incorporate two homogenization stages and recirculation loops to enhance the size reduction efficiency. Careful control of operating parameters such as applied pressure, processing time, temperature, and the number of cycles ensures that the final dispersion has a narrow and well-defined droplet size distribution [52] (Figure 3).

As reported by B. Medeiros-Neves et al. [42], two coumarin-rich extracts from *Pterocaulon balansae* were incorporated into NEs composed of a medium-chain triglyceride oil core stabilized by phospholipids. The NE with droplet sizes between 127 and 162 nm were obtained with high pressure homogenization. In addition, the antifungal activity of NEs was evaluated and showed a potential alternative for the treatment of sporotrichosis [52].

#### 4.1.2. Ultrasonication

**Figure 4:**
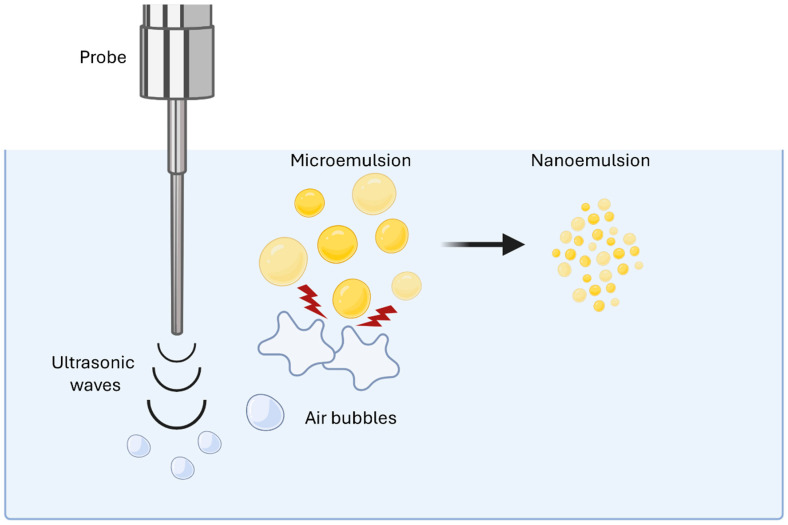
Nanoemulsion formation by ultrasonication. Created with BioRender.com.

In this technique, a probe emits ultrasonic waves into the dispersion, leading to the formation of air bubbles. These bubbles are formed when the pressure is reduced below the vapor tension of the continuous phase by the ultrasonic waves. Subsequently, the movement of the bubbles to a region of different pressure causes them to collapse, producing energy and leading to the fragmentation of dispersed microdroplets into nanodroplets (Figure 4) [53].

As reported by Y. Zhu et al. [44], ultrasonication was successfully employed to produce a novel green composite NE. In this system, glycyrrhizic acid served as a natural surfactant, while *Blumea balsamifera* oil and tea tree oil constituted the mixed oil phase. The resulting formulation demonstrated promising antibacterial and anti-inflammatory activities.

#### 4.1.3. Microfluidization

**Figure 5:**
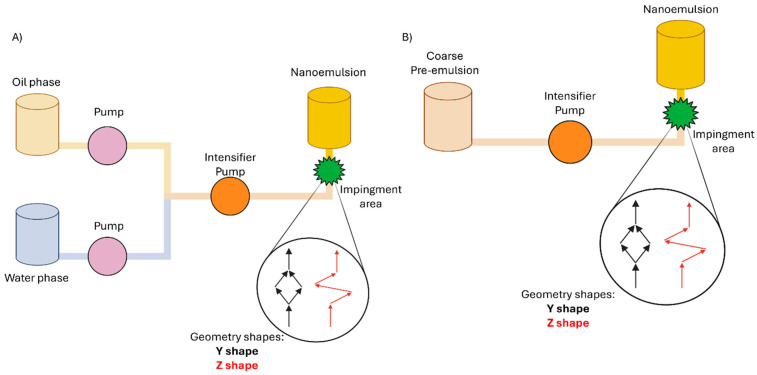
Schematic representation of feeding systems for nanoemulsion production using microfluidization. (**A**) The dual-feed system enables the independent feeding of the immiscible oil and water phases directly into the microfluidizer chamber, where the intense kinetic energy generated under high pressure drives emulsification. (**B**) The single-feed system utilizes an already formed, coarse pre-emulsion as feedstock, which is then subjected to extreme shear and impact forces within the device to achieve nanometric droplet sizes. Created with BioRender.com.

Microfluidization is a highly effective technique for producing NEs, particularly of the O/W type. The process involves combining the components (or pre-emulsion) in an impingement area and then forcing the result mixture through microchannels. This action generates very high shear stress, which is the primary mechanism for droplets size reduction. A pneumatic pump is used to apply uniform pressure, ensuring that all the material experiences the same high shear rates. This method can consistently achieve a mean droplet size lower than 0.2 μm with narrow and well-defined droplet size distribution. Key parameters influencing the final quality include the flow rate of phases, the applied high pressure, the reduced surface area and the geometry of microfluidizer system (Figure 5) [54,55].

B.J. Dukovski et al. developed and optimized a functional cationic NE for dry eye disease using chitosan and lecithin by microfluidizer system. In addition, their results indicate that preparation of the formulation may be transferable to high-energy methods other than microfluidization (Figure 5) [43].

### 4.2. Low Energy Emulsification

#### 4.2.1. Phase Inversion Composition (PIC)

**Figure 6:**
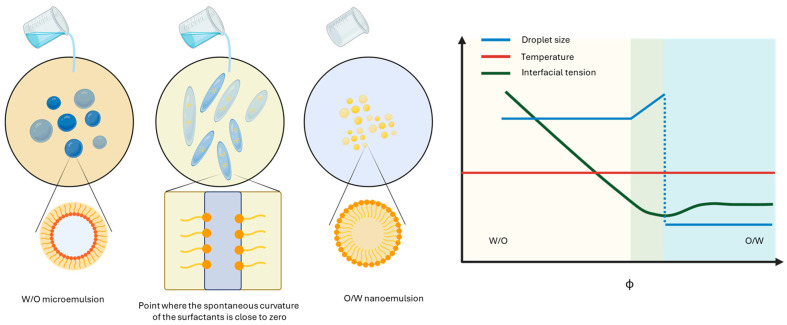
Nanoemulsion formation by phase inversion composition. The curves qualitatively illustrate the typical trend observed during PIC processing of internal phase droplet size, temperature, and interfacial tension with phase volume ratio. W/O: water-in-oil. O/W: oil-in-water. Created with BioRender.com.

This technique is commonly used for the production of O/W NEs. The crucial aspects are the selection of surfactants and the gradual addition of an aqueous water phase that, in the initial stage, behaves as the dispersed phase and progressively transitions into the continuous one [56]. The phase inversion of the emulsion is fundamentally driven by manipulating the spontaneous curvature of the surfactants. It refers to the tendency of the surfactant film to collapse, either due to the instability of insoluble surfactants or to the desorption of soluble surfactants from the interface, once the surface tension has reached saturation [57]. The goal of PIC is to reach the point where the curvature is close to zero, which corresponds to ultra-low interfacial tension, leading to a rearrangement of the oil and water phases, resulting in the desired transition. The main advantage of the PIC method is that spontaneous emulsification occurs at room temperature, in contrast to the phase inversion temperature (PIT) method (Figure 6).

In their work, Veda Prakash and Lipika Parida formulated a vitamin E-containing NE with a focus on the stirring speed during the preparation. Their results show that the increase in stirring speed led to a reduction in mean size distribution and polydispersity index [47]. Also, Ly Thi Minh Hien et al. produced a NE loaded with black pepper essential oil (*Piper nigrum* L.) by using different Tween as surfactants. Their research reveals that Tween 80 was preferable for fabricating the most stable NE [48].

#### 4.2.2. Phase Inversion Temperature (PIT)

**Figure 7:**
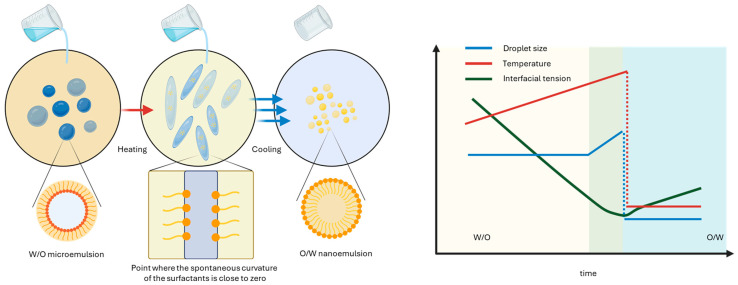
Nanoemulsion formation by phase inversion temperature. The curves qualitatively illustrate the typical trend observed during PIT processing of internal phase droplet size, temperature, and interfacial tension with time. W/O: water-in-oil. O/W: oil-in-water. Created with BioRender.com.

This emulsification process, similar to phase inversion composition (PIC), is governed by the temperature-dependent spontaneous curvature of non-ionic surfactants, which is governed by temperature changes. With an increase in temperature, the balance between the surfactant affinity for the aqueous and oil phases shifts toward a perfect balance, leading to an ultra-low interfacial tension. At temperatures close to the PIT, nonionic surfactants promote the formation of bicontinuous or lamellar structures characterized by ultra-low interfacial tension and high interfacial mobility.. When the system is rapidly cooled from this state, these transient structures break down into very small O/W droplets, which become kinetically stabilized upon curvature inversion. Final droplet size is not a simple monotonic function of temperature alone, but also depends on phase behavior, surfactant concentration, and the heating–cooling protocol (Figure 7) [56,58,59,60].

Gia Man Vu et al. prepared a saponin-stabilized NE co-encapsulating grape seed oil and rosemary essential oil, validating the PIT method as a sustainable and effective strategy for producing bioactive-loaded NEs and providing a promising foundation for future applications in health-focused food systems, cosmetics, and functional product development [45].

In their work, Xuan-Tien Le et al. successfully used PIT to produce cajeput-loaded NE. They demonstrated that this low-intensity energy method can effectively encapsulate cajeput oil with a 10% *w*/*v* surfactant concentration, while concentrations above 15% *w*/*v* are commonly reported in similar studies [46].

#### 4.2.3. Spontaneous Emulsification

**Figure 8:**
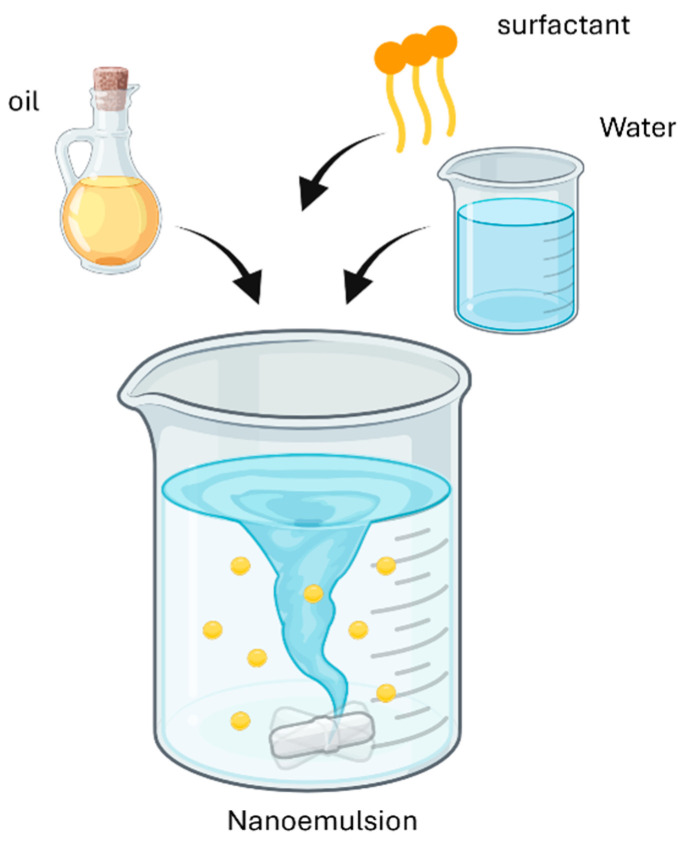
Nanoemulsion formation by spontaneous emulsification. Created with BioRender.com.

In this method, the emulsification occurs by the rapid diffusion of a surfactant and/or a co-solvent from the dispersed to the continuous phase. The entire process is not linked to the spontaneous curvature of the surfactant but to the fast movement of components in the continuous phase that creates a turbulence and, consequently, the nanodroplets (Figure 8).

In their work, Nashwa F. Tawfik et al. have successfully developed a safe and effective anti-psoriatic NE from *Artemisia monosperma* essential. The NE was obtained under mild stirring (approximately 500 rpm) by dropwise addition of distilled water to the oil phase made up of *Artemisia* oil, ethanol as a surfactant and Tween 80 as co-surfactant. A mean particle size as small as 228 nm was obtained [49].

#### 4.2.4. Vapor Condensation

**Figure 9:**
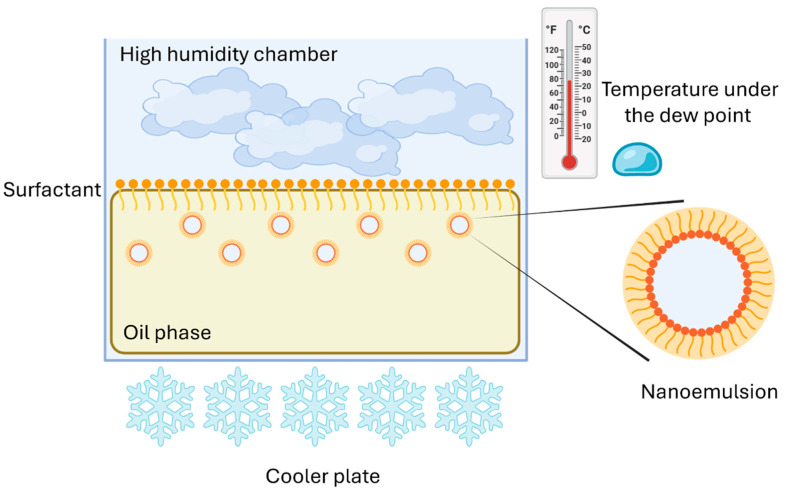
Nanoemulsion formation by vapor condensation. Created with BioRender.com.

Vapor condensation is a bottom-up assembly approach for producing NEs. The physical principle of this technique is the condensation of water phase onto the surface of oil phase. The oil phase is kept in a high humidity chamber, which is placed on a cooler plate. When the temperature of the system is reduced under the dew point, water droplets start to condensate. The droplet size distribution is strongly influenced by the processing time and the surfactant concentration in the oil phase (Figure 9). Ingrid F. Guha et al. demonstrated that nanoscale water droplets can be easily produced by this approach. Subsequently, these droplets nucleate at the oil/air interface and spontaneously disperse within the oil. Furthermore, the size distribution, along with the polydispersity index and stability, are controlled by the concentration of surfactant in the oil phase and the processing time [51].

## 5. Challenges and Optimization Strategies in Green Nanoemulsion Preparation

Despite the fast and increasing interest in the development of green NEs, the adoption of low-energy methods ramps down for high-energy methods that are currently the most commonly used. Indeed, their use is well-established in the food industry and allows for large-scale production. The main challenge in the production of green NEs is to translate the production from the laboratory to the industrial scale. The limits of their implementation are related to the high concentration of the surfactant agents to be used to reduce the interfacial tension between the two immiscible phases. Another limitation is the high energy cost related to the warming up or the fast cooling of massive volumes. So, the researchers are focusing on ingredient selection and composition optimization through the adoption and analysis of the pseudo-ternary phase diagram to identify the correct ratio of components, the possible combination of natural surfactants and co-surfactants, and/or statistical experimental design to effectively select process parameters.

Another main issue is related to the use of PUFA-rich oils, which are highly susceptible to oxidative degradation due to the high degree of unsaturation of their fatty acid chains. This represents a major limitation for the formulation and storage of NEs for pharmaceutical and nutraceutical applications [61,62]. Oxidation processes in NEs are strongly influenced by interfacial phenomena. Therefore, effective mitigation strategies for these phenomena require a combined approach that encompasses chemistry, formulation, and processing [63,64].

From a chemical perspective, the incorporation of antioxidants is a key strategy for limiting lipid oxidation. Lipophilic antioxidants such as α-tocopherol and ascorbyl palmitate are commonly used to scavenge lipid radicals in the oil phase, while amphiphilic antioxidants can be particularly effective due to their localization at the oil/water interface, where oxidation generally occurs [65,66,67,68,69]. Synergistic antioxidant systems, such as the combination of radical scavengers with metal chelators, have also been shown to improve oxidative stability compared to single-component approaches [70,71].

Formulation strategies play an equally critical role in controlling oxidation. Indeed, oil selection and blending represent a simple and effective approach. For example, PUFA-rich oils can be partially blended with less unsaturated or more oxidation-resistant oils (e.g., medium- or long-chain triglycerides) to reduce overall oxidation susceptibility. The choice of surfactant system also influences interfacial packing density and oxygen permeability, with more compact and cohesive interfacial layers generally protecting more effectively from oxidative attack [61,72]. Furthermore, secondary or multilayer NEs, obtained through polymeric or biopolymeric coatings (e.g., chitosan, proteins, or polysaccharides), can act as physical barriers that limit oxygen diffusion and the access of pro-oxidants to the oil core [73].

Processing conditions and storage practices further contribute to oxidative stability. Indeed, preparing NEs in an inert atmosphere, minimizing thermal or mechanical stress, and avoiding metal contamination during processing can significantly discourage the onset of oxidation. Regarding storage, light protection, headspace oxygen control, and the use of appropriate packaging materials are considered best practice recommendations for preserving the long-term chemical integrity of PUFA-rich NEs [74,75].

Overall, oxidative instability in PUFA-based NEs cannot be completely eliminated, but it can be effectively managed through the rational integration of antioxidant chemistry, interface design, formulation optimization, and controlled processing and storage conditions.

## 6. Pharmaceutical Applications of Green Nanoemulsions

Having discussed the formulation principles, green excipients, and preparation techniques of NEs, this section focuses on their pharmaceutical applications, highlighting how these systems translate into practical dosage forms. The application of NEs in pharmaceutical products entails other requirements that influence formulation design and technological choices. Compared to food applications, pharmaceutical NEs must follow stricter quality, safety, and reproducibility standards, including controlled excipient composition and batch-to-batch consistency.

Regulatory frameworks primarily affect the selection of excipients and acceptable processing conditions. For this reason, the present review focuses on technological and formulation aspects, highlighting how green components can be adapted to meet pharmaceutical performance and safety expectations, without aiming to provide a comprehensive regulatory analysis.

The increasing appeal of this green nanotechnology approach stems from its ability to merge pharmacological effectiveness with regulatory compliance, owing to the natural origin and established safety profile of its constituents [76]. Their remarkable diversity is due not only to the possible intrinsic bioactivity of their natural components but also to their ability to act as highly adaptable delivery systems. Moreover, their therapeutic potential covers a broad array of activities, encompassing antioxidant, anti-inflammatory, anticancer, antimicrobial, neuroprotective, cardioprotective, and wound-healing effects [77].

NEs can be formulated as many pharmaceutical dosage forms such as liquids [78,79] or gels [80,81] for multiple routes of administration, such as topical [82], oral [83], intravenous [84].

In the pharmaceutical field, O/W NEs are often used due to the affinity between the external aqueous phase and the biological environment. For instance, an ophthalmic W/O NE containing cyclosporine A was developed and approved by Food and Drug Administration (FDA). This preparation demonstrated significant effectiveness in alleviating the symptoms of dry eye syndrome, coupled with a very good safety profile [85]. In another example, propofol (DIPRIVAN^®^), a highly lipophilic sedative-hypnotic agent for intravenous administration, was formulated as an oil-in-water (O/W) lipid NEs. This preparation overcame the problem of its strong hydrophobicity, thus allowing a safe intravenous administration [86].

Differently, W/O NEs are mainly used for cutaneous applications. Recently, a W/O emulsion was formulated for the stabilization and dermal delivery of alpha-lipoic acid (ALA). This emulsion was observed to improve skin retention of the active ingredient. In particular, W/O NEs are crucial in promoting drug penetration into the skin, thereby improving the therapeutic efficacy [87].

In controlled release applications, double NEs can be used. A first noteworthy example is the preparation of a water-in-oil-in-water (W/O/W) NE based on olive oil to deliver curcumin, which is completely hydrophobic, and alpha-arbutin, which is relatively water-soluble. Curcumin and alpha-arbutin act synergistically. The formulation effectively promoted the stability of the active ingredients (>90% for three months) and led to the achievement of high anti-melanogenesis efficacy in vitro [88]. Another double NE for the oral administration of insulin has been developed. In this formulation, hydrophilic insulin preferentially localizes into the internal aqueous phase, where it is protected from gastric degradation, thereby enhancing its stability and improving intestinal absorption [89]. Drug release from NEs is mostly dictated by its partitioning from the oil phase into the surfactant layer surrounding each droplet in the dispersed phase. Subsequently, when the solubilized drug fraction migrates into the aqueous phase, nanoprecipitation occurs, which leads to an extreme increase in the specific surface area of the drug, thereby accelerating its dissolution. Importantly, this suggests that it is possible to tailor the kinetics of drug release through subtle modifications of the design and formulation phases [90].

Kumar and Mandal discuss the efficacy of surfactants in oil-in-water (O/W) NEs, highlighting how their ability to effectively lower interfacial tension aids in stabilizing emulsions with lower surfactant concentrations compared to other emulsification systems [91]. Furthermore, Handa et al. mention that while certain surfactants may require higher concentrations to achieve desired emulsification, there is potential for efficient formulation by utilizing surfactants that achieve emulsification at lower levels. Their findings point toward the necessity of considering regulatory implications related to surfactant toxicity, reinforcing the notion that lower surfactant concentrations are preferable in NEs [92]. Their findings point toward the necessity of considering regulatory implications related to surfactant toxicity, reinforcing the notion that lower surfactant concentrations are preferable in NEs [93,94].

NEs prepared with food-grade oils and green excipients, as previously discussed, represent a sustainable and biocompatible platform for the delivery of therapeutic agents [95,96,97].

Skin administration has become a particularly promising area for NE application, especially if based on green components [3]. NEs provide high drug loading capacity and the possibility of increasing the bioavailability of both hydrophilic and lipophilic drugs also maintaining a non-irritating profile on the skin layers. Indeed, the nanoscale structure promotes the diffusion of actives through the stratum corneum and increasing drug retention in the epidermis and dermis. Their moisturizing and skin compatible nature further supports their use in dermatological formulations, as well as in wound healing or localized pain management [3].

Within dermatology, NEs based on natural oil components have shown promise in the treatment of psoriasis, a chronic inflammatory skin disease characterized by excessive keratinocyte proliferation and impaired barrier function [56]. For example, Musa et al. developed cyclosporin topical NEs with excellent stability using a green matrix made of virgin coconut and nutmeg oil. This skin friendly formulation demonstrated high diffusion across rat skin ex vivo, a reduced transepidermal water loss (TEWL) and improved moisture retention [98]. Also, Sahu et al. incorporated tacrolimus with Kalonji, black seed oil, known for its intrinsic antipsoriatic activity, resulting in a marked increase in dermal bioavailability and significant clinical improvement. Another research study proposed a fully natural approach based on turmeric oil as the active component, obtaining over 70% inhibition of psoriatic inflammation in preclinical models [99,100,101]. In addition, biosurfactants, biologically derived surface-active agents, show particular promise for anti-psoriatic use owing to their biocompatibility and skin-restorative characteristics [56,102]. More recently, Khan et al. developed a dual-loaded green NE gel (FTQ-NEG) incorporating thymoquinone (TQ) and fulvic acid (FA), using kalonji oil as the lipid phase and Tween 80 as a natural surfactant. This NE demonstrated excellent safety and non-irritant properties, supporting its suitability for long-term topical application. It successfully overcame the formulation challenges associated with combining the two bioactives and also exhibited enhanced dissolution, skin permeation, and antioxidant activity. In vivo studies on psoriatic models showed significant improvements in clinical scores, histological recovery, and suppression of inflammatory cytokines (TNF-α and IL-6). compared to conventional treatments, underscoring the potential of synergistic, bioactive-based green NEs [103].

Other research studies have confirmed that NE based formulations can effectively accelerate wound healing by maintaining a moist environment, preventing microbial growth, and stimulating tissue regeneration [35,104]. When enriched with natural actives such as essential oils, curcumin, or plant-derived phenolics, these systems further enhance the repair process through their intrinsic therapeutic properties. Wound healing itself is a complex biological event involving inflammation, tissue proliferation, and remodeling. Its efficiency depends on the coordinated activity of cellular migration, angiogenesis, and collagen synthesis, which can be compromised by infection, oxidative stress, or chronic inflammation [105,106]. Also in this context, green NEs have emerged as promising systems for tissue regeneration, combining all the previously discussed advantages. Thus, to give an example, a green and multifunctional NE (BBG-NE) based on *Blumea balsamifera* oil was developed using the natural emulsifiers Bletilla striata polysaccharide and glycyrrhizic acid. This formulation exhibited excellent technological properties and strong transdermal permeation, along with enhanced antioxidant and wound-healing activity. In vivo studies on rats demonstrated rapid wound closure and almost complete skin recovery within 14 days, supported by increased fibroblast migration and modulation of inflammatory markers [107].

Beyond dermatological applications, green NEs also exhibit remarkable potential in oncology [108]. Cancer therapy is often hindered by the poor solubility, limited absorption, and non-specific toxicity of many chemotherapeutic agents. Oil-in-water NE, owing to their unique physicochemical characteristics, can effectively encapsulate lipophilic drugs, enhance bioavailability, and enable targeted delivery to tumor tissues. Moreover, these nanocarriers contribute to overcoming multidrug resistance (MDR), thereby improving both therapeutic efficacy and safety. Ongoing research continues to demonstrate their value as efficient, biocompatible, and sustainable platforms for the site-specific delivery of anticancer therapeutics [108]. A green NE (CTD-GNE) containing costunolide (CTD) a natural sesquiterpene lactone with potent anticancer activity was recently developed and optimized for lung cancer therapy [109]. Here, the formulation included α-cyclodextrin as biodegradable and biocompatible biosurfactant, to stabilize the system through self-assembly at the oil water interface, thereby improving CTD solubility and dissolution. The optimized NE, with a droplet size of around 200 nm, demonstrated enhanced cytotoxicity and pro-apoptotic activity in A549 lung cancer cells, reduced the activity of inflammatory markers with a final strong anticancer efficacy and anti-inflammatory effects, confirming its potential as a safe, eco-friendly, and effective therapeutic approach for lung cancer treatment [109].

Similarly, a honokiol-loaded green NE was developed for the treatment of glioblastoma (GBM), one of the most aggressive and recurrent brain malignancies [110]. In this study, honokiol was incorporated into a commercial lipid NE using a low-energy horizontal shaking method, consistent with green chemistry principles. The optimized formulation showed an optimal droplet size (200 nm and PDI of 0.07), high loading efficiency (~95%) and stability with suitable characteristics for intravenous administration and excellent physicochemical stability. In vitro studies demonstrated a significant reduction in glioblastoma cell viability, supporting their potential as a safe and sustainable adjuvant therapy for GBM and representing just one of several examples highlighting the therapeutic versatility of such systems [111,112].

Although the development of NE-based therapeutics has advanced considerably, only a few formulations currently under clinical investigation can be explicitly defined as green NEs. Furthermore, most ongoing trials such as those assessing ophthalmic NEs for dry eye disease (NCT03785340) and ocular graft-versus-host disease (NCT03591874) focus primarily on clinical efficacy and safety rather than on the sustainability or natural origin of their components. Similarly, another ophthalmic NE formulations containing corticosteroids, such as clobetasol propionate (NCT04249076, NCT04246801), follow conventional approaches that do not yet fully embrace the principles of green chemistry. As a result, most clinical NE studies prioritize pharmacological performance over environmental compatibility even if, as previously summarized, many experimental formulations now utilize eco-friendly and low-energy fabrication techniques, showing that sustainability and therapeutic efficacy can successfully coexist.

## 7. Thermodynamic and Physico-Chemical Insights into Nanoemulsions Stability

The thermodynamic study of NEs is essential for understanding their stability, formulation strategies, and long-term performance, especially because NEs are inherently thermodynamically unstable systems. Further, thermodynamic studies of NEs are crucial for predicting shelf life in applications such as pharmaceuticals, cosmetics, and food products.

Primary NEs tend toward phase separation due to the high interfacial energy associated with their small droplet size [113]. Their thermodynamic behavior is governed by the Gibbs energy [114]:

(1) ΔG=γΔA −TΔS

where *γ* is the interfacial tension, Δ*A* is the change in surface area, and Δ*S* the entropy change. The term *γ*Δ*A* accounts for the energy required to create new interfaces (e.g., droplet surfaces in emulsions), while *T*Δ*S* includes mixing and dispersion entropy. The formation of nano-sized droplets significantly increases surface area, which overcomes the entropic contributions that favor dispersion resulting in a positive Δ*G* that drives instability.

Despite their thermodynamic instability, NEs often display exceptional kinetic stability. This is primarily attributed to their ultra-small droplet size, which minimizes creaming and sedimentation by allowing Brownian motion to dominate over gravitational forces. Additionally, robust steric stabilization provided by surfactants and co-surfactants effectively prevents flocculation and coalescence, further enhancing their resistance to physical breakdown [115]. This kinetic stability allows NEs to persist for extended periods, leading to the description of “approaching thermodynamic stability”. Nevertheless, without continuous stabilization, phenomena such as Ostwald ripening, where larger droplets grow at the expense of smaller ones, can eventually lead to breakdown [116,117]. Overall, NEs are kinetically metastable systems but, in practical terms, they exhibit long-term kinetic stability if compared to their expected shelf lives that range from months to years, which is sufficient for pharmaceutical and industrial applications.

In addition to surfactants and co-surfactants, to enhance stability, secondary NEs are commonly employed. These systems incorporate polymeric coating that introduce additional thermodynamic interactions, such as polymer-surface binding and conformational rearrangements. Among natural and biodegradable polymers, chitosan (CS), a biopolymer derived from chitin, has gained prominence due to its biodegradability, biocompatibility, antimicrobial properties, and suitability for green formulations. Thermodynamic characterization of chitosan-based nanoparticles provides valuable insights into the energetic contributions that govern NE stability [118]. Particularly relevant in nanoparticle interactions is the entropy-enthalpy compensation. In confined environments like nanodroplets or nanopores, water exhibits modified hydrogen bonding networks and altered dielectric properties, which significantly influence solvation thermodynamics.

Researchers have increasingly employed Isothermal Titration Calorimetry (ITC) to obtain quantitative thermodynamic insights into the mechanisms underlying nanoparticles stabilization and molecular interaction [119,120,121].

Although ITC is traditionally used to study molecular interactions, it has been successfully adapted to investigate the formation and stability of secondary NEs systems in which a polymeric coating is applied to a primary emulsion core to enhance stability. A study by Fotticchia et al. [122] utilized ITC to determine key parameters such as the adsorption constant and enthalpy change (ΔH) associated with the deposition of stabilizing polymers like chitosan and poly-L-lysine onto lecithin-based NE droplets. This approach enabled the construction of a detailed thermodynamic profile of the coating process, shedding light on the intermolecular forces and structural rearrangements, including conformational changes, that contribute to the formation of a kinetic barrier against destabilization. This study demonstrated that the thermodynamic parameters governing the polymer coating process are critical for optimizing the long-term stability of NEs. It also revealed that the formation of secondary NEs is a complex, multistep thermodynamic event that extends beyond the bulk Gibbs energy of the primary emulsion. Achieving stability in these systems requires an understanding of interfacial energetics, molecular adsorption dynamics, and nanoscale structural rearrangements.

More recent work by Lagreca, E. et al. [123] explores the physicochemical validation and functional enhancements, such as mucus adhesion, of food-grade secondary oil-in-water (O/W) NEs. Using ITC, they investigated the thermodynamic behavior of NEs coated with chitosan. ITC measures the heat released or absorbed during molecular interactions. Upon addition of either pharma-grade (PG CT) or food-grade (FG CT) chitosan to negatively charged O/W NEs, an exothermic binding event was observed, followed by aggregation likely driven by electrostatic attraction. Interestingly, the enthalpy change (ΔH) was unfavorable, indicating that the process absorbed energy. However, the entropy change (Δ*S*) was favorable, suggesting increased disorder. This favorable entropy contribution outweighed the enthalpic cost, resulting in a spontaneous interaction (Δ*G* < 0).

Both PG CT and FG CT exhibited similar thermodynamic profiles, indicating that the degree of chitosan modification did not significantly influence the interaction. This points to electrostatic attraction as the dominant stabilizing mechanism. By analyzing these thermodynamic parameters, researchers can predict the optimal chitosan concentration for coating, improve the stability of the final NE product and design more effective formulations for pharmaceutical and food applications.

Beyond the thermodynamic framework described above, a deeper mechanistic understanding of interfacial chemistry is essential to explain the formation, stability, and structural evolution of NEs. In general, interfacial tension (γ) at the oil–water interface, decreases markedly upon surfactant adsorption [124]. This reduction in γ lowers the energy barrier for droplet breakup and directly affects ΔG in Equation (1), facilitating the formation of nano-sized droplets.

Dynamic tensiometry and microfluidic studies demonstrate that the magnitude and rate of γ reduction are governed by surfactant adsorption/desorption kinetics, where rapid diffusion and fast interfacial adsorption generate a responsive interfacial layer capable of accommodating the intense deformation imposed during high-energy emulsification [125]. Conversely, slower adsorption kinetics limit droplet refinement and reduce resistance to coalescence, particularly when interfacial layers remain incompletely formed under shear [126].

The molecular structure of surfactants plays a decisive role in determining interfacial packing and the resulting spontaneous curvature of the interfacial film. Surfactants with long or bulky hydrophobic tails promote lower curvature (favoring W/O structures), whereas shorter or more flexible hydrophobic groups and larger hydrophilic headgroups typically support positive curvature encouraging O/W NEs [127].

Numerous studies show that ethoxylation degree, unsaturation in the hydrophobic tail, and headgroup charge density modulate interfacial packing constraints, bending elasticity, and ultimately the minimum attainable droplet size [128,129,130,131].

Oil polarity is another critical mechanistic determinant. More polar oils such as medium-chain triglycerides exhibit lower interfacial tension and often promote more efficient surfactant adsorption compared to highly non-polar long-chain triglycerides [132]. Oil polarity also influences Ostwald ripening susceptibility by modulating oil solubility in the continuous phase. Oils with higher aqueous solubility generate steeper chemical potential gradients, thereby increasing ripening rates [133,134]. Selecting oils with low polarity or blending with a small fraction of insoluble oils (e.g., long-chain triglycerides or squalene) can mitigate this destabilization pathway [133,135].

Collectively, these mechanistic insights highlight that NE performance is governed not only by thermodynamic driving forces but also by the intricate interplay between surfactant molecular architecture, interfacial dynamics, and oil phase properties.

## 8. Rheology of Nanoemulsions

Owing to their nanoscale droplet size and high interfacial area, NEs exhibit a mechanical behavior that differs fundamentally from those of conventional macroemulsions. Compared to macroemulsions, NEs exhibit significantly higher elasticity, which is particularly relevant for applications such as cosmetics, food, and pharmaceuticals, where texture and consumer perception are critical. The rheological properties of NEs can be tuned by controlling the dispersed phase volume fraction (Φ) and droplet size, and by the addition of salt, depletion agents, and polymers that can physically associate among themselves or with NE droplets [2,136,137]. Furthermore, processing parameters, such as the number of high-pressure homogenization passes, can induce a transition from a flowing fluid to a slowly relaxing or gel-like system due to droplet rupture and jamming [138].

As discussed above, NE inherent instability presents a major challenge for their use in many technological and industrial applications, where long-term stability is essential [139]. For this reason, understanding the mechanisms governing emulsion stability and identifying strategies to enhance it remains a central focus of current research. Key factors such as droplet size, surfactant concentration, oil volume fraction, and processing conditions are routinely investigated to optimize emulsion performance [140,141].

In this context, a recent study [103] demonstrated that rheological measurements can serve as a powerful tool for assessing the structural integrity of NEs over time. Using oscillatory shear tests, the authors monitored how the viscoelastic moduli, G′ (storage modulus) and G″ (loss modulus), vary with strain and frequency. Their results show that, in concentrated NEs, G′ is much higher than G″ within the linear viscoelastic (LVE) region, indicating a predominantly elastic, gel-like structure. G′ remains constant at low strains, reflecting a stable internal network. At the yield strain, however, G′ decreases sharply while G″ exhibits a pronounced peak, thus evidencing a structural relaxation as the network begins to break and the material transitions toward flow.

Macroemulsions behave very differently. They exhibit significantly lower moduli and only a weak peak in G″, pointing to a less organized, loosely connected microstructure. This more fluid-like character makes them more susceptible to instability phenomena such as creaming or coalescence. In contrast, the tighter packing and stronger gel-like character of NEs is directly linked to their enhanced long-term stability [2].

The study also shows that oil concentration strongly influences rheological behavior. Increasing oil content leads to higher G′ and G″, with NEs displaying a marked jump in moduli once a critical oil volume fraction is exceeded. This behavior corresponds to the formation of densely jammed networks, which substantially increase the shear modulus. Beyond this threshold, further increases in oil concentration have minimal effect, indicating that the system has reached a maximum packing limit. Finally, surfactant concentration also plays a decisive role. Higher surfactant levels restrict droplet mobility and can drive the system into a kinetically trapped state, thereby improving stability by limiting coalescence and enhancing depletion interactions [142].

## 9. Conclusions and Future Perspectives

Green nanoemulsions represent a promising and increasingly sophisticated platform for improving the solubility, stability, and bioavailability of hydrophobic therapeutic and nutraceutical products. The shift from conventional formulations to biocompatible, pharmaceutical, or food-grade systems is in line with the increasing interest in sustainable and safe materials. A major strength of food-grade nanoemulsions is their ability to combine drug solubilization with intrinsic therapeutic benefits, particularly in the case of oils rich in bioactive lipids or antioxidants.

However, despite significant advances in formulation strategies and therapeutic applications, several challenges remain that must be addressed to fully exploit the potential of these carriers.

One major challenge is rational formulation design. While plant-derived surfactants and food-grade oils improve safety and sustainability, their composition variability can be detrimental for reproducibility and biological performance. A clearer understanding of interfacial phenomena, and excipient-drug interaction is essential, particularly for balancing small droplet size, long-term kinetic stability, and controlled release.

Furthermore, while many short-term in vitro and in vivo studies demonstrate increased bioaccessibility and favorable pharmacokinetics, long-term toxicological assessments and mechanistic investigations are still limited. In-depth studies on the interaction between oil type, surfactant composition, digestion, and drug release are needed to rationalize formulation choices beyond empirical optimization. In addition, stability remains a complex issue. Indeed, lipid oxidation, aggregation, and phase transitions during storage can compromise performance, particularly for nutraceuticals and essential oils. Advances in the design of synergistic antioxidant systems and the selection of customized excipients can mitigate degradation pathways, but a deeper molecular understanding of oxidative mechanisms is still needed.

Manufacturing and scalability are a major challenge too. Indeed, the translation of laboratory-scale emulsification to industrial milieu may compromise droplet size, uniformity, or stability. Thus, tailored approaches integrating low-energy methods, green solvents, and waste-minimizing strategies will be critical to enable scalable production while retaining the environmental advantages.

Toxicological evaluation is still another main point. Although natural excipients are generally perceived as safe, systematic long-term studies addressing cumulative exposure, allergenicity, dermal penetration pathways, and potential immunomodulatory effects are still limited. Standardized in vitro and in vivo models would provide support for regulatory approval.

Overall, this review highlights that green nanoemulsions insofar are not merely a conceptual extension of conventional systems but rather represent a realistic and technologically mature platform for pharmaceutical applications, if formulation design, excipient choice, and processing strategies are carefully tailored. While significant challenges remain in terms of reproducibility, stability, and large-scale translation, the convergence of sustainable materials and advanced formulation approaches points at green nanoemulsions as promising candidates for future pharmaceutical development.

## Figures and Tables

**Table 1:** Composition, oxidation, and solubility parameters of food-grade oil.

Oil	SFA/MUFA/PUFA (% *w*/*w*)	OSI (h, 110–120 °C)	PV (meq O_2_/kg)	Solubilization Parameters
Coconut oil	85–92/6–8/<2	30–40	0–10	Solubilization capacity
MCT oil	>95/~0/~0	>40	<5	Medium–High
Olive oil	12–15/70–80/5–10	6–12	2–20	Medium
Soybean oil	12–15/25–30/55–60	1–7	1–5	High
Sunflower oil	8–10/20–30/55–65	1–4	4–16	High
Fish oil	25–35/20–30/30–45	<1–2	5–15	Medium

SFA, saturated fatty acids; MUFA, monounsaturated fatty acids; PUFA, polyunsaturated fatty acids; OSI, Oxidative Stability Index; PV, Peroxide Value.

**Table 2:** Classification of surfactants.

Surfactant	Chemical Class	HLB Range	CMC	pH/Ionic Sensitivity	Regulatory Notes
Whey protein isolate (WPI)	Protein	~8–10 (apparent)	Not defined	Sensitive near isoelectric point (pH~4–5); affected by ionic strength	Widely used in pharmaceutical systems; not listed as standard excipient in major pharmacopeias
Soy protein isolate (SPI)	Protein	~8–10 (apparent)	Not defined	pH- and salt-sensitive; reduced stability near pI	GRAS; no monograph in USP/Ph.Eur.
Soy lecithin	Phospholipid	~4–8	Not clearly defined	Moderate sensitivity to pH and ions due to charged headgroups	GRAS; listed in USP–NF and Ph.Eur.
Quillaja saponins	Saponins	Not defined	Low (reported micellization)	Sensitive to ionic strength; stable across pH 3–8	Natural extract; regulatory limits depend on purity and region; no official monograph
Gum arabic	Polysaccharide	Not defined	Not applicable	Low sensitivity;	Pharmaceutical excipient; listed in USP–NF and Ph.Eur

HLB range, CMC, pH/ionic sensitivity, regulatory limits. HLB: Hydrophilic–Lipophilic Balance; CMC: Critical Micelle Concentration; pI: isoelectric point. For high-molecular-weight biopolymers, HLB and CMC are approximate or not strictly defined and are reported when available from literature.

**Table 3:** Some main techniques used for the production of nanoemulsions. nd: not determined (data not available).

Methods	Techniques	Physical Principles	Active Molucule	Technological Characterization	Stability	Activity	References
High-energy	High pressure homogenization (HPO)	High speed and high shear stress	Coumarins (*P. balansae* extracts)	Mean size: from 127 to 162 nm PDI: <0.15 ζ: from −21 to −39 mV	nd	Antifungal activity against *S. schenckii* strains	[42]
			Ibuprofen	Mean size: 175.1 ± 1.1 nm PDI: 0.127 ± 0.013 ζ: 24.6 ± 0.4 mV	30 days	Anti-inflammatory	[43]
	Ultrasonication	Cavitation	*Blumea balsamifera* Oil Tea Tree Oil Glycyrrhetinic acid	mean size: 160.01 nm PDI: 0.125 ζ: −50.94 mV	120 days	Antibacterial activity against *Escherichia coli, Staphylococcus aureus*, and *Pseudomonas aeruginosa*. Anti-inflammatory activity in Adjuvant-Induced Arthritis rats	[44]
	Microfluidization	Geometry of apparatus and surface area	Ibuprofen	Mean size: 175.1 ± 1.1 nm PDI: 0.127 ± 0.013 ζ: 24.6 ± 0.4 mV	30 days	Anti-inflammatory	[43]
Low-energy	Phase inversion temperature (PIT)	Temperature changing and spontaneous curvature of surfactant	Grape seed oil Rosemary essential oil	Mean size:109.6 ± 2.186 nm PDI: 0.124 ± 0.008 ζ: −18.7 ± 0.943 mV	30 days	Antioxidant	[45]
			Cajeput essential oil	Mean size: 20.5 nm PDI: 0.45 ζ: nd	120 days	nd	[46]
	Phase inversion composition (PIC)	spontaneous curvature of the surfactants	Vitamin E	Mean size: from 5.77 to 11.89 nm PDI: <0.35 ζ: from 14.3 to 10.6 mV	30 days	nd	[47]
			Black pepper essential oil	Mean size: 9.60 nm PDI: 0.324 ζ = nd	5 weeks	nd	[48]
	Spontaneous emulsification	Self-assembling	*Artemisia monosperma* essential oil	Mean size: 228 nm PDI:0.406 ζ: −9.4 mV	3 months	Anti-psoriatic	[49]
			Dapagliflozin	Mean size: 96.64 nm PDI: 0.402 ζ: −24.2	6 month	nd	[50]
	Vapor condensation (VC)	Condensation	nd	Mean size: 215 nm PDI: 0.2 ζ: nd	nd	nd	[51]

## Data Availability

No new data were created or analyzed in this study. Data sharing is not applicable to this article.
